# Persistent Unilateral Nephrolithiasis: Do 24-h Urine Parameters Account for It?

**DOI:** 10.3390/jcm15176731

**Published:** 2026-08-30

**Authors:** Orel Hemo, Miki Haifler, Asaf Shvero, Nir Kleinmann, Dorit E. Zilberman

**Affiliations:** Department of Urology, Sheba Medical Center, Faculty of Medicine, Tel Aviv University, Ramat Gan 5262000, Israeldorit.zilberman@sheba.health.gov.il (D.E.Z.)

**Keywords:** nephrolithiasis, unilateral, stone formation, urine composition

## Abstract

**Background:** Bilateral nephrolithiasis is typically attributed to systemic metabolic abnormalities reflected in 24 h urine studies. Yet, some individuals form stones exclusively in one kidney over long-term follow-up, while the contralateral kidney remains unaffected. This study aimed to determine whether differences in urinary metabolites can explain persistent unilateral stone formation. **Methods:** We retrospectively reviewed our 2011–2024 nephrolithiasis database and analyzed 24 h urine compositions obtained under regular diet and daily activity conditions. Patients were classified into three groups: bilateral stone formers (BSF), unilateral stone formers (USF), and past unilateral stone formers (PUSF)—the latter comprising patients whose stone-forming kidney was either resected or non-functioning, with urine reflecting the contralateral, stone-free kidney. **Results:** The study included 618 patients in the BSF group, 22 in the USF group, and eight in the PUSF group. The median follow-up duration was 83 months. After correction for multiple comparisons, the only difference between the USF and BSF groups that remained significant was higher urinary calcium in the USF group (269 vs. 184 mg/24 h; adjusted *p* = 0.03). The PUSF group had lower creatinine and uric acid than the other groups, and lower citrate than the USF group (adjusted *p* < 0.05); the differences in oxalate did not remain significant after correction. **Conclusions:** Standard 24 h urine parameters, which reflect combined output from both kidneys, did not account for persistent unilateral nephrolithiasis. This suggests that local renal, molecular, or genetic factors, rather than systemic metabolic differences, may underlie this phenomenon.

## 1. Introduction

Bilateral nephrolithiasis may be considered a result of urine metabolic abnormalities detected simultaneously in both kidneys [1]. The high risk of stone formation is attributed to either an excess or deficiency of certain urine metabolites [2]. Accordingly, hypercalciuria, hyperuricosuria, hypocitraturia, and low urine volume, together or individually, are possible causes of renal stone formation [2].

A high urine sodium level is considered a risk factor for hypercalciuria, given the linear relationship between Na^+^ and Ca^2+^ excretion within the nephron unit and the resultant higher-than-expected Ca^2+^ excretion due to high dietary Na^+^ intake [3].

However, there are cases in which only one kidney forms stones persistently over long-term follow-up, while the contralateral kidney always remains stone-free. This enigma raises the question of whether urine metabolite differences between kidney pairs could explain this phenomenon.

Three small studies sought to explore this issue [1,4,5].

These findings were inconclusive due to the small sample sizes, very short duration of urine collection (no longer than 12 h), and urine collection during urological surgery, which may alter metabolite levels through anesthetics or stress effects.

In the present study, we sought to reveal whether there are metabolite differences between the 24 h urine collections of kidney pairs of various stone formers and, if so, whether they can serve as an explanation for the persistent unilateral stone formation phenomenon.

## 2. Materials and Methods

Following approval by the Sheba Medical Center ethics (Helsinki) committee (SMC-4478-17) which waived the need for informed consent for this retrospective and anonymized study, we reviewed our registry database of all patients with nephrolithiasis seen at our outpatient clinic.

### 2.1. Patient Selection and Definition of Laboratory Values

As part of their follow-up and as recommended by the most recent American Urological Association guidelines [6], the study participants had undergone a metabolic workup that included blood tests and 24 h urine collection for volume, creatinine, sodium, potassium, calcium, uric acid, oxalate, and citrate levels. The study included recurrent urinary tract stone formers, defined as more than two stones documented during the follow-up period, who were older than 18 years, had normal urinary tract anatomy, and had a minimum follow-up of 24 months. Patients were included only if they had at least one metabolic evaluation, conducted 6–8 weeks after stone lithotripsy, regardless of the results. For classification as unilateral stone formers (USF or PUSF), more than two stones had to be documented on the same side, while the contralateral kidney remained stone-free throughout the entire follow-up period.

The exclusion criteria included patients younger than 18 years of age, patients with cystinuria, a history of upper tract urothelial carcinoma, ileal conduit, ileal ureter, reconstructed or augmented urinary bladder, anatomic anomalies (e.g., horseshoe kidneys or pelvic kidneys), obstructive ureteral pathology such as congenital or acquired ureteral stenosis/stricture, as well as patients with a congenital single kidney.

A urine volume of <2000 mL/day was defined as low and a urine sodium level of >200 mmol/day was defined as high; hypercalciuria was set at >250 mg/day (women) or >300 mg/day (men); hyperuricosuria was set at >750 mg/day (women) or >800 mg/day (men); hyperoxaluria was set at >40 mg/day; and hypocitraturia was set at <320 mg/day, in accordance with established guideline definitions [6]. Urine collection was carried out at least 2 months following stone fragmentation or stone expulsion and under a regular diet and daily activities. Patients were instructed to perform the test on a weekday, rather than on weekends when their food and drinking habits may fluctuate. Additionally, patients were advised to maintain their customary diet, drinking, and exercise habits. They were also advised to discontinue, for one week prior to urine collection, the use of drugs that might affect urine metabolic parameters, such as diuretics and calcium supplements.

### 2.2. Methods

The documented demographic data included age, sex, body mass index, the presence of hypertension, diabetes mellitus or dyslipidemia, follow-up length, operated side, and nephrolithiasis surgical history.

Patients were examined either annually or bi-annually at our outpatient clinic, and any change in their stone status was recorded. Stone burden and laterality were assessed at each visit by non-contrast computed tomography and/or renal ultrasonography; classification as a unilateral stone former required that the contralateral kidney remain stone-free on all available imaging throughout follow-up.

We compared 24 h urine metabolites between bilateral stone formers (the BSF group) and unilateral stone formers (the USF group).

The BSF group comprised patients who formed stones in both kidneys either simultaneously or in different periods, and the 24 h urine collection reflected both kidneys.

The USF group comprised patients in whom a new stone was documented on imaging, always on the same side, at least one year after the previous documented event (renal colic or surgery for stone fragmentation), confirming persistent ipsilateral recurrence over time.

Their 24 h urine collection also reflected both the stone-forming kidney and the stone-free kidney.

The past unilateral stone formers (the PUSF group) comprised patients who had met the above criteria for unilateral stone formation while both kidneys were intact and functioning, and whose stone-forming kidney was subsequently resected or lost function (≤10%) during follow-up. These two subgroups were analyzed together because, irrespective of whether the affected kidney had been surgically removed or had ceased to function, the 24 h urine collection in each case reflects excretion from a single, contralateral, stone-free kidney—the condition of interest for the present study. In all PUSF patients, the metabolic evaluation was performed during routine outpatient follow-up once the patient was stone-free, rather than at a predefined time point after loss of renal function.

For the entire cohort, urine collection for metabolic evaluation was performed only after stone-lithotripsy (or kidney resection in the PUSF group), when the patients were stone free. Importantly, in the BSF and USF groups the 24 h urine collection reflects the combined output of both kidneys and therefore represents whole-body, not kidney-specific, metabolite excretion. Only in the PUSF group does the collection reflect a single, isolated kidney. Accordingly, the between-group comparisons in this study are exploratory and cannot directly establish whether the stone-forming and stone-free kidneys differ metabolically.

### 2.3. Statistical Analysis

Statistical analysis was performed using IBM SPSS software, version 26.0 (Armonk, NY, USA: IBM Corp, 2019) and R v. 4.3.1 (R Core Team, 2021). R: A language and environment for statistical computing. (R Foundation for Statistical Computing, Vienna, Austria). Data are presented as frequencies (percentages) for categorical variables or as median (interquartile range [IQR]) for continuous variables. Continuous variables were compared with the Kruskal–Wallis rank test, and a pairwise analysis was performed with the Wilcoxon rank-sum test if they were found to be significant.

Categorical variables were compared with the Fisher exact test.

Multiple comparisons were controlled using the Benjamini–Hochberg false discovery rate. Effect sizes for pairwise comparisons are reported as the rank-biserial correlation coefficient (r). Because 24 h creatinine differed between groups, a sensitivity analysis was performed in which each solute was expressed as a ratio to urinary creatinine, thereby accounting for possible differences in collection completeness and muscle mass. Statistical significance was defined as *p* < 0.05 for 2-sided tests.

## 3. Results

Our registry database included data collected between 2011 and 2024, and 648 patients fulfilled the study inclusion criteria and were included in the statistical analysis. The median follow-up period for the entire group was 83 [59–107] months.

There were 618 patients in the BSF group, 22 patients in the USF group and eight patients in the PUSF group. Out of the PUSF group, six had undergone resection of the stone-forming kidney and two had a non-functioning kidney on renal-scan.

Overall, the groups were similar in their demographic characteristics (Table 1).

The metabolite levels of 24 h urine collections were inconsistent across the three groups (Table 2).

Pairwise comparisons with FDR correction (Table 3) localized these differences. Compared with the BSF group, the USF group had higher urinary calcium (269 vs. 184 mg/24 h; adjusted *p* = 0.034; r = 0.33), whereas the apparent differences in uric acid and oxalate between the USF and BSF groups did not remain significant after correction. The PUSF group differed mainly from the other two: it had lower creatinine (vs. BSF and USF; both adjusted *p* = 0.010, r = −0.75 and −0.86, respectively), lower uric acid (vs. USF, adjusted *p* = 0.010, r = −0.79; vs. BSF, adjusted *p* = 0.034, r = −0.51), and lower citrate than the USF group (adjusted *p* = 0.034, r = −0.65). No pairwise oxalate comparison remained significant after correction. Urine volume, pH, sodium, and potassium did not differ significantly among the groups.

In a sensitivity analysis normalizing solute excretion to urinary creatinine (Supplementary Table S1), none of the between-group differences remained significant after FDR correction. In particular, the lower creatinine, uric acid, and citrate observed in the PUSF group were attenuated and no longer significant, indicating that these differences were largely attributable to lower overall creatinine excretion in this older, predominantly female, single-kidney subgroup, rather than to a stone-specific metabolic profile.

## 4. Discussion

The persistent unilateral renal stone-forming phenomenon remains poorly understood, given the assumption that the causes of stone formation should affect both kidneys.

Urine metabolite disturbances traditionally serve as a cornerstone of nephrolithiasis pathophysiology [2].

We hypothesized that unilateral urine metabolite anomalies were the cause of persistent unilateral renal stone formation. To test this hypothesis, we would have ideally performed 24 h urine collections from each of the two kidneys under standard dietary and daily activities and compared the results. This is not feasible in practice. Alternatively, we could have performed this collection in patients admitted for stone fragmentation, but low patient compliance would have been inevitable and understandable, in addition to raising the level of stress associated with the procedure. Therefore, we used our registry database and the recorded 24 h urine collections of the patients in the USF, BSF and PUSF groups in an attempt to extrapolate the results to determine whether there is a difference in urine metabolite anomalies that could explain persistent stone formation exclusively in the same kidney.

Because the 24 h collections in the BSF and USF groups reflect combined bilateral excretion rather than the output of the affected kidney alone, the following comparisons should be interpreted as exploratory and hypothesis-generating rather than as direct evidence of kidney-specific metabolic differences. Although urine volumes and pH levels were comparable, several differences were observed on omnibus testing. After pairwise comparison with FDR correction, the only difference between the USF and BSF groups that remained significant was the higher urinary calcium in the USF group; the apparent differences in uric acid, oxalate, and citrate did not survive correction. This is consistent with prior observations that the balance between urinary calcium and citrate, rather than either metabolite alone, influences stone risk [7].

The PUSF group exhibited a distinct metabolic pattern, with lower creatinine and uric acid than the other groups and lower citrate than the USF group, whereas calcium and oxalate did not differ significantly. The substantially lower urinary creatinine in the PUSF group most likely reflects its older age, higher proportion of women, and single functioning kidney—all associated with lower creatinine excretion—rather than a stone-related mechanism. Consistent with this, when solutes were indexed to creatinine, the apparent metabolic differences in the PUSF group were no longer evident. Because pre-nephrectomy (or pre-functional-loss) urine was not available, we cannot determine whether this pattern reflects a true physiological adaptation to the loss of one kidney, pre-existing baseline differences, or a selection effect within this small subgroup. A comparable post-nephrectomy reduction in citrate was previously reported by Danilovic et al. [8] who found hypocitraturia in patients with struvite or calcium-oxalate stones. These findings highlight the complexity of metabolic alterations in kidney stone disease and underscore the need for individualized treatment strategies that account for both the type and number of kidneys, as well as their functional status.

Three small studies have previously tried to address the issue of persistent unilateral stone formation.

Eisenberg et al. [5] collected urine samples from patients with nephrolithiasis who underwent unilateral percutaneous nephrolithotomy (PNL).

Urine was collected for 12 h via Foley catheters from both the treated kidney via nephrostomy tube and the untreated kidney from 14 patients with unilateral nephrolithiasis and 17 patients with bilateral nephrolithiasis. Contrary to our findings, there was less excretion of calcium and uric acid among the unilateral cases compared to the bilateral cases, and, similar to our findings, less excretion of sodium was observed in the unilateral group.

A weakness of that study was its small sample size (a total of 31) and the timing of urine collection (12 h post-operatively), which may have reflected both post-surgical stress and anesthetic influence and consequently altered the results.

Later on, Sichani et al. [4] collected 24 h urine samples from 22 patients with unilateral staghorn stones and 56 patients with bilateral staghorn stones who were candidates for PNL.

There was no difference in metabolite levels between the groups; however, significantly higher cystine levels were observed in the bilateral staghorn stone group. The main pitfall of this study was that the authors understandably could not isolate urine from the other kidney, precluding the possibility of addressing the mechanism of unilateral stone formation.

In an attempt to do so, Ziemba et al. [1] collected urine samples from 13 patients during retrograde upper urinary tract surgery, and obtained paired samples from eight of them. Five of those eight patients had at least one unilateral metabolic abnormality.

Again, the mechanism responsible for unilateral stone formation could not be elucidated, this time due to the small study sample and the single urine sample.

The results of the present study did not reveal any substantial correlation between urine metabolite levels and unilateral renal stone formation.

Our findings support Le et al. [9], who found no correlation between profound hypocitraturia and the extent or laterality of nephrocalcinosis.

They are also consistent with reports that 1.1–35% of stone formers have no metabolic abnormalities in 24 h urine collections [10,11,12].

In contrast, metabolic abnormalities were found in non-stone formers who, regardless, never formed any stones [13].

Alternative mechanisms for unilateral renal stone formation have been suggested, such as sleep posture [14], different urine microbiome [15], as well as vascular injury causing hypo- or hyperperfusion that, in turn, led to the induction of stress-induced, crystal-binding molecules [9].

We believe that the answer most likely lies at the molecular or genetic level. Nephrolithiasis has a well-recognized heritable component, and local differences in gene or protein expression between the paired kidneys—which would not be reflected in systemic 24 h urine chemistry—may explain why stone formation remains confined to one side. This possibility warrants dedicated genetic and molecular investigation.

On a practical level, our findings suggest that whole-body 24 h urine metabolite levels do not, on their own, explain why stones recur in one kidney rather than both. This observation concerns stone laterality specifically and does not diminish the established role of 24 h urine testing in guiding individualized metabolic therapy for stone prevention [16].

In fact, given that potassium citrate is indicated in the treatment of hypocitraturia, hypercalciuria and hyperuricosuria [2,6], repeated stone formation is sufficient reason to implement treatment with this regimen.

The present study provides a reasonable perspective regarding the 24 h urine composition in persistent unilateral and bilateral stone formers.

The strengths of the current study include its large sample size compared to previous studies, the long follow-up duration (mandatory mainly for validating the persistent unilateral stone formers group), the collection of urine under normal dietary and lifestyle activities, and the full-day urine collections as opposed to the segmental or half-day collections reported in previous studies.

Its limitations include its retrospective nature and the relatively small sample size of the unilateral groups, especially the PUSF subgroup; these comparisons are therefore underpowered, and the PUSF findings in particular should be regarded as exploratory. A further important limitation is the absence of stone composition data. Because dusting is the predominant lithotripsy technique at our institution, stone fragments were not available for compositional analysis in the great majority of patients, which could have helped clarify whether the metabolic differences observed between the groups track with stone type, and its absence therefore limits the mechanistic interpretation of our metabolic findings. In addition, our method of concluding on both kidneys is indirect, and we were unable to obtain urine solely from the contralateral kidney to avoid invasive procedures. There is a need for future research to evaluate urine metabolites in each urinary system in order to enhance our findings.

Furthermore, since 24 h urine collection was not obtained before the resection of the stone-forming kidney in the PUSF group, we could not compare 24 h urine collection pre- and post-nephrectomy. In addition, in the PUSF group the 24 h urine was collected during routine follow-up at a variable interval after nephrectomy or loss of renal function; because the compensatory physiology of a solitary functioning kidney may evolve over time, this variability could have influenced the observed urine values.

Nevertheless, extrapolating the results we obtained provides some insight into the urine metabolite compositions in persistent bilateral and unilateral stone formers and suggests that metabolite abnormalities may not be directly associated with the risk of stone formation.

## 5. Conclusions

In this exploratory comparison, differences in whole-body 24 h urine composition did not consistently distinguish persistent unilateral from bilateral renal stone formation, although the indirect nature of the sampling precludes a definitive conclusion regarding kidney-specific metabolism. Finding a solution for this enigma at the molecular/genetic level may facilitate the search for the dynamics that underlie unilateral kidney stone disease.

## Figures and Tables

**Table 1:** Demographic data.

	Total	BSF	USF	PUSF	*p* Value
	N = 648	N = 618	N = 22	N = 8	
Age at presentation (years), median (IQR)	54 (44, 64)	54 (43.75, 64)	51 (46, 61.5)	65.5 (55.75, 67)	0.141
Male sex, N (%)	483 (74.5)	464 (75.2)	16 (72.7)	3 (37.5)	0.05
Creatinine *, mg/dL, median (IQR)	0.89 (0.75, 1)	0.9 (0.75, 1)	0.81 (0.69, 0.94)	0.82 (0.68, 0.93)	0.67
BMI, median [IQR]	27.8 (24.5, 31.1)	27.7 (24.5, 31.1)	28.7 (25.1, 33.6)	29.6 (21.3, 30.1)	0.562
Hypertension, N (%)	213 (33.1)	201 (32.8)	9 (40.9)	3 (37.5)	0.704
Diabetes mellitus, N (%)	150 (23.1)	139 (22.8)	8 (36.4)	3 (37.5)	0.217
Dyslipidemia, N (%)	177 (27.8)	166 (27.3)	9 (40.9)	2 (25)	0.372
Follow-up length (months), median [IQR]	83 (59, 107)	83 (59, 107)	95 (71, 122)	71 (53,92)	0.33

BSF = Bilateral Stone Formers; USF = Unilateral Stone Formers; PUSF = Past Unilateral Stone Formers; N = Number; IQR = Interquartile Range. * Creatinine level 6–8 weeks after stone lithotripsy.

**Table 2:** Metabolite levels of 24 h urine collections.

Characteristic	Overall, N = 648 ^1^	BSF, N = 618 ^1^	USF, N = 22 ^1^	PUSF, N = 8 ^1^	*p*-Value ^2^
Volume (cc)	1700 [1250, 2300]	1700 [1238, 2300]	1675 [1300, 2275]	1750 [1550, 1875]	>0.9
Creatinine	1576 [1200, 1900]	1587 [1200, 1900]	1656 [1335, 1925]	950 [896, 1057]	**0.005**
PH	5.5 [5, 6]	5.5 [5, 6]	5.75 [5.37, 6]	5.5 [5.12, 6.6]	0.941
Sodium	170 [123, 232]	170 [123, 232]	158 [131, 222]	142 [98, 184]	0.6
Potassium	62 [48, 84]	62 [48, 84]	56 [49, 80]	64 [58, 75]	0.7
Calcium	187 [118, 288]	184 [117, 284]	269 [216, 337]	123 [69, 280]	**0.030**
Uric acid	594 [466, 760]	597 [466, 766]	623 [570, 705]	446 [393, 499]	**0.033**
Oxalate	32 [26, 43]	32 [26, 43]	36 [31, 46]	24 [18, 36]	**0.042**
Citrate	494 [292, 710]	494 [292, 709]	669 [389, 970]	218 [194, 448]	**0.030**

^1^ Median [IQR]. ^2^ Kruskal–Wallis rank sum test. BSF = Bilateral Stone Formers; USF = Unilateral Stone Formers; PUSF = Past Unilateral Stone Formers. Bold indicates significance.

**Table 3:** Pairwise comparisons for the metabolites significant on omnibus (Kruskal–Wallis) testing.

Metabolite	Comparison	Median A	Median B	*p* (unadj.)	*p* (FDR)	r
**Creatinine**	BSF vs. USF	1600	1657	0.529	0.529	+0.08
	BSF vs. PUSF	1600	950	0.002	**0.009**	−0.75
	USF vs. PUSF	1657	950	0.002	**0.009**	−0.86
**Calcium**	BSF vs. USF	184	269	0.013	**0.034**	+0.33
	BSF vs. PUSF	184	123	0.367	0.424	−0.20
	USF vs. PUSF	269	123	0.116	0.146	−0.41
**Uric acid**	BSF vs. USF	596	623	0.515	0.529	+0.09
	BSF vs. PUSF	596	446	0.014	**0.034**	−0.51
	USF vs. PUSF	623	446	0.002	**0.009**	−0.79
**Oxalate**	BSF vs. USF	32	36	0.082	0.123	+0.24
	BSF vs. PUSF	32	24	0.069	0.115	−0.37
	USF vs. PUSF	36	24	0.035	0.074	−0.53
**Citrate**	BSF vs. USF	495	669	0.093	0.127	+0.23
	BSF vs. PUSF	495	218	0.045	0.084	−0.44
	USF vs. PUSF	669	218	0.012	**0.034**	−0.65

BSF = Bilateral Stone Formers; USF = Unilateral Stone Formers; PUSF = Past Unilateral Stone Formers. Bold indicates significance after FDR correction.

## Data Availability

The datasets used and/or analyzed during the current study are available from the corresponding author upon reasonable request.

## Supplementary Materials

The following supporting information can be downloaded at: https://www.mdpi.com/article/10.3390/jcm15176731/s1. Table S1. Sensitivity analysis: 24-h urinary solutes expressed as a ratio to urinary creatinine.

## Abbreviations

BSF	Bilateral Stone Formers
USF	Unilateral Stone Formers
PUSF	Past Unilateral Stone Formers
