# Supplementary material for: Persistent Unilateral Nephrolithiasis: Do 24-h Urine Parameters Account for It?
Source: J Clin Med. 2026 Aug 30;15(17):6731. doi: 10.3390/jcm15176731 (PMC13566434; doi:10.3390/jcm15176731)
Supplement: Supplementary file 1 [file jcm-15-06731-s001.zip › jcm-4482590-supplementary.pdf]

Supplementary Table S1. Sensitivity analysis: 24-h urinary solutes expressed as a ratio to urinary creatinine.

Ratio (mg/g Cr)	Comparison	Median A	Median B	p (unadj.)	p (FDR)	r
**Calcium/Cr**	BSF vs USF	126	190	0.023	0.277	+0.32
	BSF vs PUSF	126	114	0.812	0.886	-0.06
	USF vs PUSF	190	114	0.378	0.648	-0.26
**Uric acid/Cr**	BSF vs USF	395	421	0.665	0.797	+0.06
	BSF vs PUSF	395	453	0.116	0.536	+0.37
	USF vs PUSF	421	453	0.256	0.614	+0.33
**Oxalate/Cr**	BSF vs USF	21	23	0.134	0.536	+0.22
	BSF vs PUSF	21	20	0.948	0.948	+0.02
	USF vs PUSF	23	20	0.507	0.760	-0.20
**Citrate/Cr**	BSF vs USF	322	432	0.208	0.614	+0.19
	BSF vs PUSF	322	242	0.609	0.797	-0.13
	USF vs PUSF	432	242	0.313	0.625	-0.32

BSF, bilateral stone formers; USF, unilateral stone formers; PUSF, past unilateral stone formers; Cr, creatinine.
